# XGEM: Predicting Essential miRNAs by the Ensembles of Various Sequence-Based Classifiers With XGBoost Algorithm

**DOI:** 10.3389/fgene.2022.877409

**Published:** 2022-03-28

**Authors:** Hui Min, Xiao-Hong Xin, Chu-Qiao Gao, Likun Wang, Pu-Feng Du

**Affiliations:** ^1^ College of Intelligence and Computing, Tianjin University, Tianjin, China; ^2^ Institute of Systems Biomedicine, Department of Pathology, School of Basic Medical Sciences, Beijing Key Laboratory of Tumor Systems Biology, Peking-Tsinghua Center of Life Sciences, Peking University Health Science Center, Beijing, China

**Keywords:** essential miRNA, CART, XGBoost, sequence features, ensemble classifier

## Abstract

MicroRNAs (miRNAs) play vital roles in gene expression regulations. Identification of essential miRNAs is of fundamental importance in understanding their cellular functions. Experimental methods for identifying essential miRNAs are always costly and time-consuming. Therefore, computational methods are considered as alternative approaches. Currently, only a handful of studies are focused on predicting essential miRNAs. In this work, we proposed to predict essential miRNAs using the XGBoost framework with CART (Classification and Regression Trees) on various types of sequence-based features. We named this method as XGEM (XGBoost for essential miRNAs). The prediction performance of XGEM is promising. In comparison with other state-of-the-art methods, XGEM performed the best, indicating its potential in identifying essential miRNAs.

## Introduction

MicroRNAs (miRNAs) are functional non-coding RNAs of ∼22 nt in length. miRNAs are involved in regulating gene expressions (He and Hannon, 2004) in animals and plants. They have diverse expression patterns and regulate many biological processes, including cell proliferation (Cao et al., 2022), cell differentiation (Martin et al., 2016), cell apoptosis (Zhang et al., 2019), fat metabolism (Nematbakhsh et al., 2021), and development of animals and plants (Zhang et al., 2018). They are also related to many complex diseases (Wojciechowska et al., 2017), including many types of tumors (Zhang et al., 2007; Lee and Dutta, 2009; Fridrichova and Zmetakova, 2019).

*lin-4* (Lee et al., 1993) was the first miRNA to be discovered, followed by *let*-7 (Reinhart et al., 2000). The regulatory roles of miRNAs have been widely studied (Bartel, 2004, 2018). Although miRNAs are small in length, their cellular role is important. Knocking out or knocking down some miRNA genes will result in lethal or infertile phenotypes (Bartel, 2018). These miRNAs genes are thought to be essential for the organism to live or develop. With the progress of miRNA gene annotations, many computational methods were developed to find miRNA genes in the genome (Wang et al., 2019). However, this resulted in many annotated miRNA genes in the database with little or no functional understanding (Bartel, 2018; Ru et al., 2019). As a basis toward the understanding of gene cellular functions, a gene should be determined if it is essential or not (Zeng et al., 2018; Campos et al., 2019).

In the context of miRNA genes, there are two categories of methods for identifying essential miRNAs: experimental methods and computational predictions. The experimental methods usually perform gene knockout or gene expression knockdown experiments on animal or plant models. By observing the phenotypes, the essentiality of the gene in question will be determined (Larrimore and Rancati, 2019). For example, Ahmed et al. (2017) reported that the *miR*-7a-2 is an essential miRNA gene by knocking out the *miR*-7a-2 gene in the mouse genome to observe the result that it caused infertility. Since the experimental methods are inevitably time-consuming and labor-intensive, computational predictions are always considered as alternative approaches or, at least, beneficial supplements. Computational prediction methods usually combine machine learning algorithms with statistical features of genomic sequences and structures to construct classifiers. Currently, there is no genome-wide clear set of essential miRNA genes. Therefore, constructing such machine learning-based predictors for essential miRNA genes is still a challenging task. As far as we know, only a handful of studies tried to predict essential miRNAs.

Ru et al. (2019) carried out a study in computationally predicting essential miRNAs. They collected 85 essential miRNAs from the literature (Bartel, 2018). By compensating 88 non-essential miRNAs from their own random selection, they presented a benchmarking dataset for computationally predicting essential miRNAs. They achieved a promising result by applying a simple voting scheme in the ensemble of multiple classifiers. Song et al. (2019) collected 77 essential miRNAs from the same literature (Bartel, 2018). They proposed the miES method based on the logistic regression algorithm. Yan et al. (2020) developed a third method based on the same 77 essential miRNAs, namely, PSEM, for the prediction of essential miRNAs in the mouse genome.

In this study, we applied the XGBoost (extreme gradient boosting) method (Chen and Guestrin, 2016) with classification trees to construct our predictor on various sequences and structural features. By optimizing features and parameters, we achieved better prediction performances than existing studies. We named our method as XGEM (XGBoost for essential miRNAs). We provided genome-wide prediction results in mice as a supplemental annotation to the mouse genome.

## Materials and Methods

### Experimental Data

We considered the dataset from Ru’s work (Ru et al., 2019), which contains 85 essential and 88 non-essential pre-miRNA sequences. We also obtained the dataset of miES (Song et al., 2019) and PESM (Yan et al., 2020) work, which contains 77 essential miRNAs. To compose a working dataset, we randomly picked up 77 non-essential miRNAs as negative samples for the miES and PESM dataset. We noted the former dataset as Ru’s dataset and the latter dataset as the miES-PESM dataset. Ru’s dataset was used for training and testing the XGEM method, while the miES-PESM dataset was used only for performance comparison.

### Feature Extraction Methods

Five sequence feature extraction methods were incorporated in our work. They are *k*-mer frequencies, sequence mismatch features, subsequence features, PseDSSPC (pseudo-distance structure status pair composition), and triplet compositions. BioSeq-Analysis 2.0 (Liu et al., 2019) and repRNA (Liu et al., 2016b) were used to generate these features. Although the algorithms for generating these features have been elaborated in various works of the literature (Chen et al., 2015, 2018; Liu et al., 2016a, 2019; Zhang et al., 2021), we briefly described them here for the convenience of readers.

Given an RNA sequence *R* with length *l*, it can be noted as follows:

$$R=r_{1}r_{2}...r_{l},$$ (1)

where *r*_*i*_ (*i* = 1, 2, 3, … *l*) ∈ {A, C, G, U} is the *i*-th residue in *R*.

The *k*-mer frequencies are the appearance frequency of 4^*k*^ type’s *k* consecutive nucleotides. The sequence *R* is separated into *l*–*k* + 1 *k*-mers, which are *r*_1_ *r*_2_ … *r*_*k*_, *r*_2_ *r*_3_ … *r*_*k*+1_, … , and *r*_*l*-*k*+1_ *r*_*l*-*k*+2_ *r*_*l*_. We noted the *k*-mer frequency as a vector of 4^*k*^ dimensions (Wei et al., 2014), which can be noted as follows:

$$F_{1}\left(k\right)={\left[\begin{array}{cccc} f_{1,1} & f_{1,2} & \cdots & f_{1,4^{k}} \end{array}\right]}^{T},$$ (2)

where *f*_1,*i*_ (*i* = 1, 2, … , 4^*k*^) is the frequency of the *i*-th type of *k*-mer, and *T* is the transpose operator.

The mismatch feature is proposed by Leslie et al*.* as an alternative method of *k*-mer frequencies (Leslie et al., 2004). The method considers inaccurate matching and calculates the number of occurrences of *k* consecutive nucleotides that differ by at most *m* mismatches (*m* = 0, 1, … , *k*-1). We define the mismatch feature vector as follows:

$$F_{2}\left(k,m\right)={\left(\begin{array}{cccc} \sum_{j=0}^{m}c_{1,j} & \sum_{j=0}^{m}c_{2,j} & \cdots & \sum_{j=0}^{m}c_{4^{k},j} \end{array}\right)}^{T},$$ (3)

where *c*_*i*,*j*_ (*i* = 1, 2, … , 4^*k*^ and *j =* 0, 1, … , *m*) is the number of occurrence of the *i* ^th^ type *k*-mer in sequence *R* with exactly *j* mismatches*.*

The subsequence feature is a method that allows non-continuous matching, which considers more matching situations (Lodhi et al., 2002). The value of the feature vector is determined by the number of occurrences of the subsequence and a decay factor *δ* ∈ [0, 1]. The subsequence feature vector of sequence *R* is defined as follows:

$$F_{3}\left(k,m\right)={\left(\begin{array}{cccc} \sum_{a_{1}}\delta^{l\left(a_{1}\right)} & \sum_{a_{2}}\delta^{l\left(a_{2}\right)} & \cdots & \sum_{a_{4^{k}}}\delta^{l\left(a_{4^{k}}\right)} \end{array}\right)}^{T},$$ (4)

where *a*_*i*_ (*i* = 1, 2, … , 4^*k*^) is a subsequence in *R* with possibly non-contiguous matching to the *i* ^th^ type of *k*-mer, and *l* (*a*_*i*_) a length function can be defined as follows:

$$l\left(a_{i}\right)=\{\begin{array}{cc} 0 & a_{i}\text{ is a contiguous matching of the }i-\text{th type of }k-\text{mer}\\ \left|a_{i}\right| & \text{otherwise} \end{array}.$$ (5)

|.| is the operator to calculate the length of a string.

Triplet feature is a combination of the primary sequence and secondary structural information of RNA. It was proposed by Xue et al.( 2005). By using the ViennaRNA package (Lorenz et al., 2011), we can estimate the secondary structure of *R* as follows:

$$S=s_{1}s_{2}s_{3}\cdots s_{l},$$ (6)

where *s*_*i*_ (*i* = 1, 2, .., *l*) ∈ { ' (', ')', '.' } denotes the secondary structure status of the *i* ^th^ residue. The “ (‘ and ’)” represent the residue in a pairing status, while "." represents the unpairing status. By ignoring the difference between “ (‘ and ’)”, there are eight possible structural statuses of a triplet. Combining the structural status and the centered nucleotide of a triplet, 32 types of possible structural triplets can be obtained. Therefore, a 32-dimensional vector can be constructed to describe the appearance frequency of all structural triplets, which can be noted as follows:

$$F_{4}={\left[\begin{array}{cccc} f_{4,1} & f_{4,2} & \cdots & f_{4,32} \end{array}\right]}^{T},$$ (7)

where *f*_4,*i*_ (*i* = 1, 2, … , 32) is the normalized frequency of the *i*-th structural triplet.

PseDSSPC was proposed by Liu *et al.* (Liu et al., 2016a). It represents the RNA sequence by considering both local and global information of secondary structures. Let *t*_*i*_ (*i* = 1, 2, … , *l*) ∈ {A, C, G, U, A-U, U-A, G-C, C-G, G-U, and U-G} be the structural status of the *i*-th residue, where A, C, G, and U represent the four types of unpaired residues, while A-U, U-A, G-C, C-G, G-U, and U-G represent the six paired status. For every *t*_*i*_, its free energy *e* (*t*_*i*_) can be calculated. We first computed the raw appearance frequency of each of the 10 structural status, which can be noted as *g*_5,1_, *g*_5,2_, … *g*_5,10_. Given a parameter *d*, we can calculate the appearance frequency of all structural status pairs with a distance in the range [1, *d*]. These can be noted as *g*_5,11_, *g*_5,12_, … , *g*_5,110_, g_5,111_, *g*_5,112_, … , *g*_5,210_, … , *g*_5,10+(*d*-1)100+1_, *g*_5,10+(*d*-1)100+2_, … , *g*_5,10+100*d*_. After that, with a lag parameter *λ*, correlation coefficients can be computed for the serial of free energy values. The *k* ^th^ tier correlation coefficient can be defined as follows:

$$g_{5,10+100d+k}=\frac{1}{l-k}\sum_{i=1}^{l-k}{\left[e\left(t_{i}\right)-e\left(t_{i+k}\right)\right]}^{2},$$ (8)

where *k* = 1, 2, … , *λ*.

With all aforementioned definitions, we can construct PseDSSPC features as follows:

$$F_{5}={\left[\begin{array}{cccc} f_{5,1} & f_{5,2} & \cdots & f_{5,10+100d+\lambda } \end{array}\right]}^{T},$$ (9)

where *T* is the transpose operator,

$$f_{5,i}=\{\begin{array}{cc} \frac{g_{5,i}}{1+d+w\sum_{k=10+100d+1}^{10+100d+\lambda }g_{5,k}} & 1\leq i\leq 10+100d\\ \frac{wg_{5,i}}{1+d+w\sum_{k=10+100d+1}^{10+100d+\lambda }g_{5,k}} & 10+100d+1\leq i\leq 10+100d+\lambda \end{array},$$ (10)

and *w* is a balancing parameter.

#### XGBoost With Classification Trees as Base Classifiers

We used CART (Classification and Regression Trees) with the Gini index as the purity function (Grajski et al., 1986) to create base classifiers in this work. Given a sample set *D*, the Gini function is defined as follows:

$$G\left(D\right)=\sum_{i=1}^{k}p_{i}\left(1-p_{i}\right)=1-\sum_{i=1}^{k}p_{i}^{2},$$ (11)

where *k* is the number of classes in the set, and *p*_*i*_ is the proportion of the *i* ^th^ class.

Considering an attribute α, the set D is divided into several subsets according to different values of α. The purity at this branching node is defined as follows:

$$I\left(D,\alpha \right)=\sum_{j=1}^{v}\frac{\left|D_{j}\right|}{\left|D\right|}G\left(D_{j}\right),$$ (12)

where *v* is the number of subsets, *D*_*j*_ is the *j*-th subset, and |.| is the cardinal operator of a set.

XGBoost (Chen and Guestrin, 2016) was used to create ensembles for boosting performances of classification trees.

### Performance Measures

Four statistics, including accuracy (*Acc*), precision (*Pre*), recall (*Rec*), and F1-score (*F*), are used to quantitively describe the performance of our method. They are defined as follows:

$$Acc=\frac{TN+TP}{FN+FP+TN+TP},$$ (13)

$$Pre=\frac{TP}{TP+FP},$$ (14)

$$Rec=\frac{TP}{TP+FN},$$ (15)

$$F=\frac{2Pre\cdot Rec}{Pre+Rec},$$ (16)

where *TP*, *TN*, *FP*, and *FN* are the number of true positives, true negatives, false positives, and false negatives, respectively. We also used the area under the receiver operating characteristic (AUROC) curve to measure the performance of our model.

### Parameter Calibration

We used a grid search strategy with leave-one-out cross-validation to find the optimal parameters. For *k*-mer features, we scanned *k* = 1, 2, 3, 4, 5, and 6. For mismatch features, we scanned *k* = 1, 2, 3, 4, 5, and 6 and *m* ∈ [0, *k*-1] with a step of 1. For subsequence features, we scanned *k* = 2, 3, and 4, and *δ* ∈ [0.1, 0.9] with a step of 0.1. In PseDSSPC, we scanned *d* ∈ [1,10] with a step of 1, *λ* ∈ [1, 20] with a step of 1 and *w* ∈ [0.1, 0.9] with a step of 0.1.

Different combinations of parameter values in CART and XGBoost are explored. We adjusted three parameters in the CART algorithm, including the randomness of branching (*S*), the maximum depth (*D*), and the maximum number of features (*M*). We scanned *S* ∈ [“best”, “random”], *D* ∈ [3,10] with a step of 1 and *M* ∈ [3, *n*] with a step of 1, where *n* is the number of sample features. We adjusted *S*, *D*, and *M* in order; when the former parameters are being scanned, the latter ones are set as default values. The best value of the former is applied to the latter parameter adjustment. We adjusted four parameters in XGBoost, including the number of trees (*T*), the learning rate (*R*), the maximum depth of trees (*D*), and the regularization parameter (*λ*). We scanned *T* ∈ [50, 500] with a step of 10, *R* ∈ [0.1, 0.5] with a step of 0.02, *D* ∈ [3, 10] with a step of 1, and *λ* ∈ [0, 2] with a step of 0.1. Similar strategies to the CART parameter optimization were applied.

### System Implementation

**FIGURE 1:**
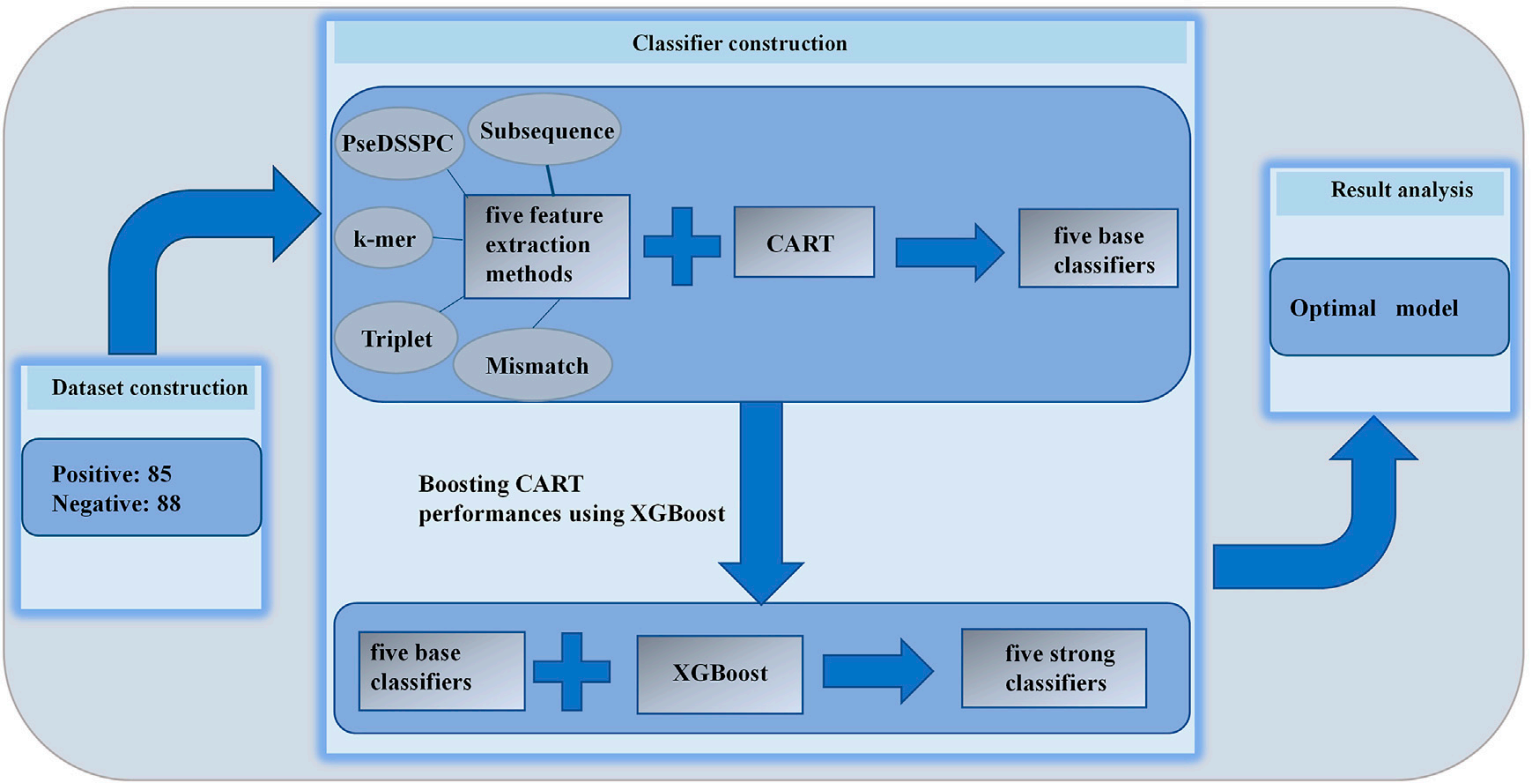
Overall flowchart of our method.

The CART and XGBoost algorithms are implemented using Python with the scikit-learn package. The whole flowchart of this work is illustrated in Figure 1.

## Results and Discussions

### Performance Analysis by CART

**FIGURE 2:**
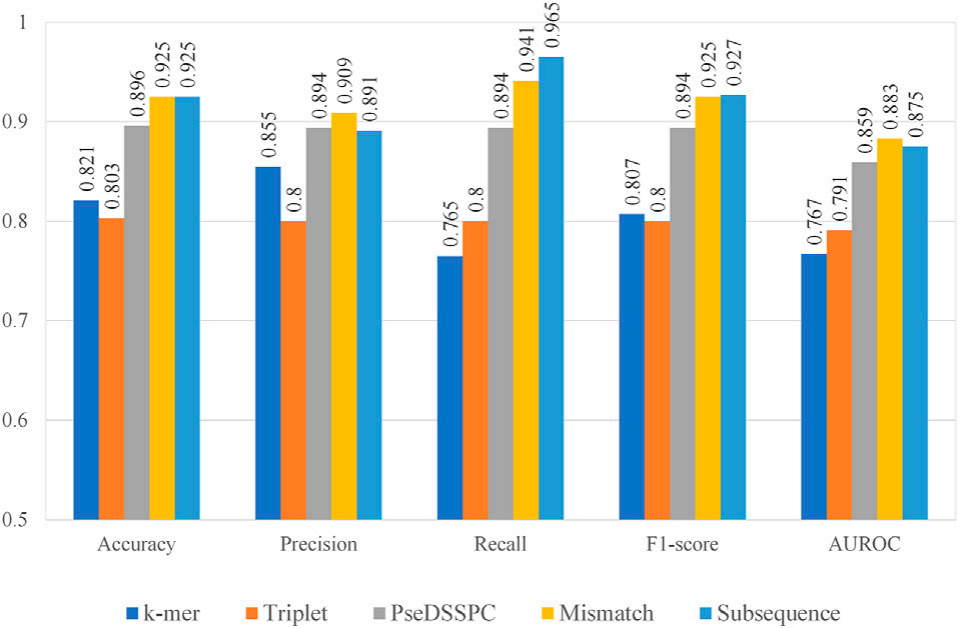
Prediction performances using five different types of features with CART.

We combined each of the five feature extraction methods with CART. We optimized the parameters of each kind of features. The best performances of each type of features can be found in Figure 2. The evaluation was performed on Ru’s dataset. Leave-one-out cross-validation protocol was applied on each type of features. The entire record of the parameter optimization process can be found in Supplementary Tables S1–S5.

From Figure 2, the subsequence features seem to have the best performances among the five. It has the highest or second to the highest value in terms of all performance measures. On the contrary, the performances of *k*-mer features and triplet features seem not as high as the others. The *k*-mer features have lowest performance values in terms of recall and the AUROC. The triplet features have the lowest performance values in terms of accuracy, precision, and F1-score. However, the precision value of *k*-mer and the recall value of triplet features are still competitive, which make them still worth a further boosting analysis. It should be noted that the PseDSSPC features, which by design would preserve most of the sequence information, did not give outstanding performances. This may be the result of the CART classifier, which cannot sufficiently utilize the information in this form.

**FIGURE 3:**
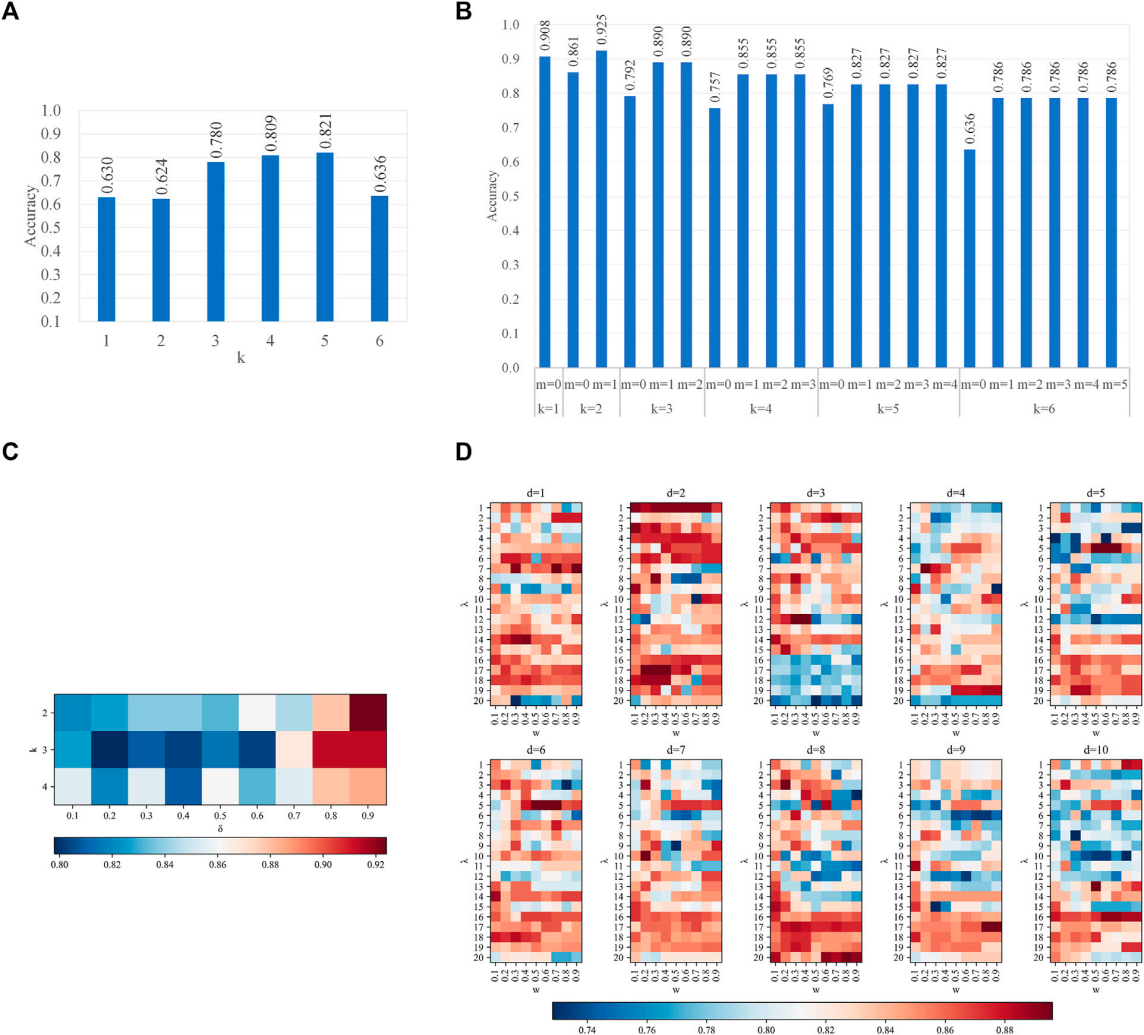
Parameter effects on CART with different types of features. Parameters of features are scanned **(A)**. *k*-mer features **(B)**; mismatch features **(C)**; subsequence features **(D)**; PseDSSPC features. In **(A)** and **(B)**, the vertical axis is the accuracy in leave-one-out cross-validation. In **(C,D)**, the heat color represents the accuracy in leave-one-out cross-validation. The optimized parameter is *k* = 5 for the *k*-mer features, *k* = 2 and *m* = 1 for the mismatch features, *k* = 2 and *δ* = 0.9 for the subsequence features, and *d* = 5, *λ* = 5, and *w* = 0.5 for the PseDSSPC features.

**FIGURE 4:**
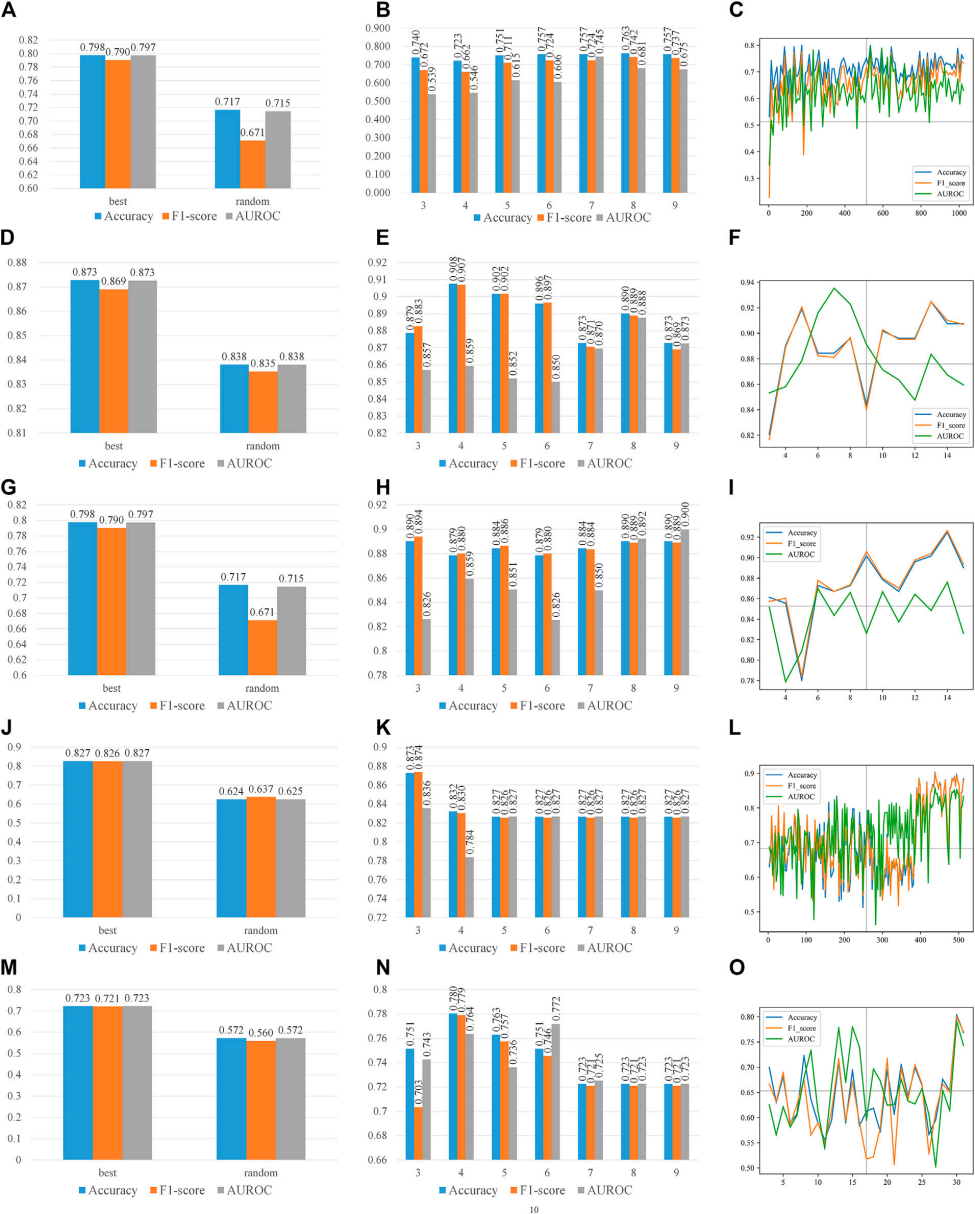
Parameter effects on CART with different types of features. Parameters of CART are scanned. The accuracy, F1-score, and AUROC are presented in each panel. **(A)**, **(B)**, and **(C)** are scanning CART parameters *S*, *D*, and *M* on *k*-mer features, respectively, and **(D)**, **(E)**, and **(F)** are scanning CART parameters *S*, *D*, and *M* on mismatch features, respectively; **(G)**, **(H),** and **(I)** are scanning CART parameters *S*, *D*, and *M* on subsequence features, respectively; **(J)**, **(K)**, and **(L)** are scanning CART parameters *S*, *D*, and *M* on PseDSSPC features, respectively; **(M)**, **(N)**, and **(O)** are scanning CART parameters *S*, *D*, and *M* on the triplet features, respectively. The best parameter for *k*-mer features is *S* = ‘best’, *D* = 8, and *M* = 490. The best parameter for mismatch features is *S* = ‘best’, *D* = 4, and *M* = 13. The best parameter for subsequence features is *S* = ‘best’, *D* = 3, and *M* = 14. The best parameter for PseDSSPC features is *S* = ‘best’, *D* = 3, and *M* = 460. The best parameter for the triplet features is *S* = ‘best’, *D* = 4, and *M* = 30.

With the optimal features, we analyzed the effect of different parameters in two steps. The first step is to analyze the effect of parameters in features, the latter one for the parameters in CART. When we performed the first step analysis, the parameters in the second step were fixed as their optimal values and vice versa. Figure 3 recorded the effects of parameters on all type of features. On all four types of features, which have at least one parameter each, the prediction accuracy peaks at some combinations of parameters, while it valleys with other combinations. Therefore, the parameters of features affect the performances. Figure 4 recorded the effects of CART parameters on all types of features. The peaks of the parameter *D* are the most significant. Although the parameter *M* causes the most fluctuation on performances, it is generally a random oscillation without easily observable patterns. Due to limited figure panel spaces, we only present a subset of performance measures in the figures. As we have mentioned, a comprehensive and quantitative record can be found in Supplementary Tables S1–S5.

### Boosting CART Performances Using XGBoost

We applied XGBoost on the CART classifiers with each of the five types of features. The parameters of XGBoost are optimized to get the best AUROC. Leave-one-out cross-validations were performed on Ru’s dataset. The prediction performances of the best boosted classifiers are listed in Table 1.

**TABLE 1:** Performance of the five strong classifiers.

Models	Accuracy (%)	Precision (%)	Recall (%)	F1-Score (%)	AUROC^a^ (%)
*k*-mer	82.7	80.9	84.7	82.8	86.4
Mismatch	96.0	94.3	97.6	96.0	96.4
Subsequence	93.1	94.1	94.1	94.1	97.3
PseDSSPC	90.8	91.6	89.4	90.4	94.8
Triplet	80.9	80.9	80.0	80.4	85.3

a AUROC is the area under a receiver operating characteristic curve.

According to Table 1, the subsequence features achieved 97.3% AUROC after boosted by XGBoost, which is the highest AUROC among all five models. However, its performances in terms of other measures are not as high as the mismatch features. The mismatch features achieved the best values in accuracy, precision, recall, and F1-score. Therefore, the mismatch features and the subsequence features with XGBoost are better choices than the other three for predicting essential miRNAs.

**FIGURE 5:**
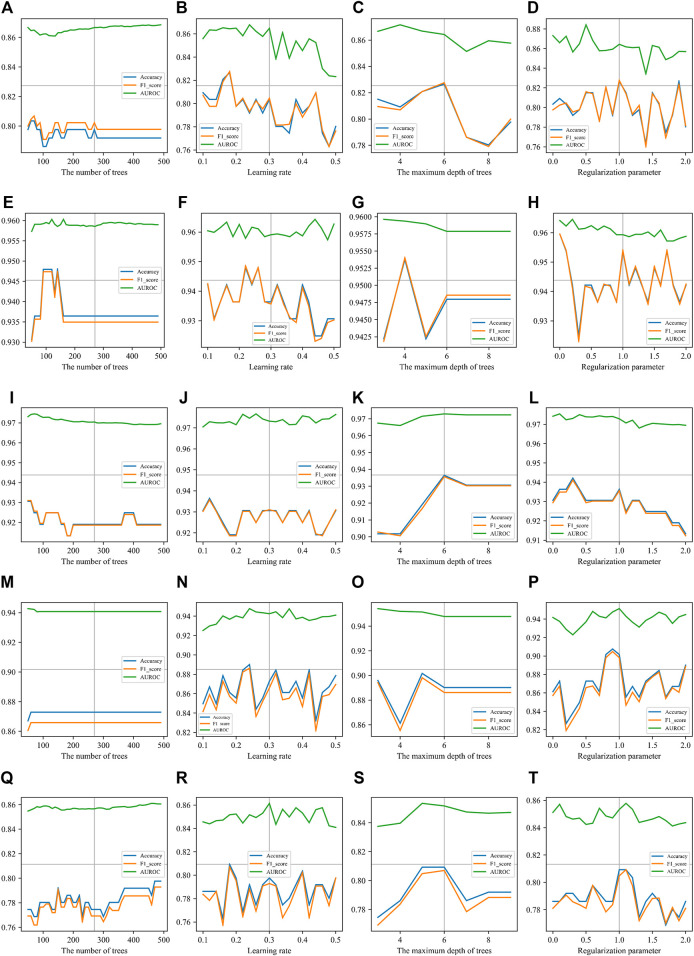
Parameter effects on XGBoost with different types of features. Parameters of XGBoost are scanned. The accuracy, F1-score, and AUROC are presented in each panel. The number of trees **(T)**, the learning rate **(R)**, the maximum depth of trees **(D)**, and the regularization parameter (*λ*) are scanned on each type of sequence features. **(A), (B)**, **(C)**, and **(D)** are scanning parameters on *k*-mer features. The best parameter values are *T* = 60, *R* = 0.18, *D* = 6, and *λ* = 1. **(E)**, **(F)**, **(G)**, and **(H)** are scanning parameters on mismatch features. The best parameter values are T = 80, R = 0.22, D = 4, and λ = 0. **(I), (J)**, **(K)**, and **(L)** are scanning parameters on subsequence features. The best parameter values are T = 50, R = 0.12, D = 6, and λ = 0.3. **(M)**, **(N)**, **(O)**, and **(P)** are scanning parameters on Pse-DSSPC features. The best parameter values are T = 60, R = 0.24, D = 5, and λ = 0.9. **(Q) (R)**, **(S)**, and **(T)** are scanning parameters on triplet features. The best parameter values are T = 500, R = 0.18, D = 5, and λ = 1.

Similar to the analysis on non-boosted CART classifiers, we performed an analysis to see the results with different XGBoost parameter values. Figure 5 gives the details of all results when the parameters are adjusted. Due to limited space in the figure panels, we only presented three performance measures. Full records can be found in Supplementary Table S6 . All curves in Figure 5 show that the AUROC is just slightly affected by the parameters of XGBoost. The accuracy and F1-score ride the same tides when parameters are turned. Because of the theoretical relationship between F1-score and the accuracy, this observation indicated that the classifier is boosted in a balanced manner by XGBoost. This is an expected behavior of a good boosting framework on an informative and balanced training dataset.

### Independent Dataset Test

We selected mismatch features with XGBoost and subsequence features with XGBoost as the optimal models. We tested the feasibility of the two models in predicting potential essential miRNAs. We collected 16 mouse pre-miRNAs from various works of the literature, which had no overlap with our training dataset, as an independent testing dataset (Supplementary Table S7). Among them, eight were essential, and the others were non-essential. On this testing dataset, the mismatch features with XGBoost achieved 90.6% AUROC. The subsequence features with XGBoost achieved 81.2% AUROC. Therefore, we believe that the mismatch features with XGBoost is the one best choice for predicting essential miRNAs. We named this method XGEM (XGBoost for essential miRNAs).

### Genome-wide Prediction

We downloaded all 1,234 mouse pre-miRNA sequences from the miRbase (Kozomara et al., 2019). The 85 essential miRNAs and 88 non-essential miRNAs in the training dataset were removed. The 16 sequences in the testing dataset were also removed, leaving 1,045 sequences with unknown essentiality. XGEM was applied to create predictions for all of them. The results are recorded in Supplementary Table S8. It can provide guidance for the study of miRNA biological function experiments. It should be noticed that XGEM was trained on balanced datasets. However, the real world is highly imbalanced. Therefore, false positives are inevitable in the prediction results. But this does not diminish the value of the results as the prediction shrinks the range of potential essential miRNAs to a much smaller scale, which is exactly the purpose of computational predictions.

### Comparison With State-of-the-Art Methods

We compared XGEM to all existing state-of-the-art methods, including Ru’s work (Ru et al., 2019), miES (Song et al., 2019), and PESM (Yan et al., 2020).

**FIGURE 6:**
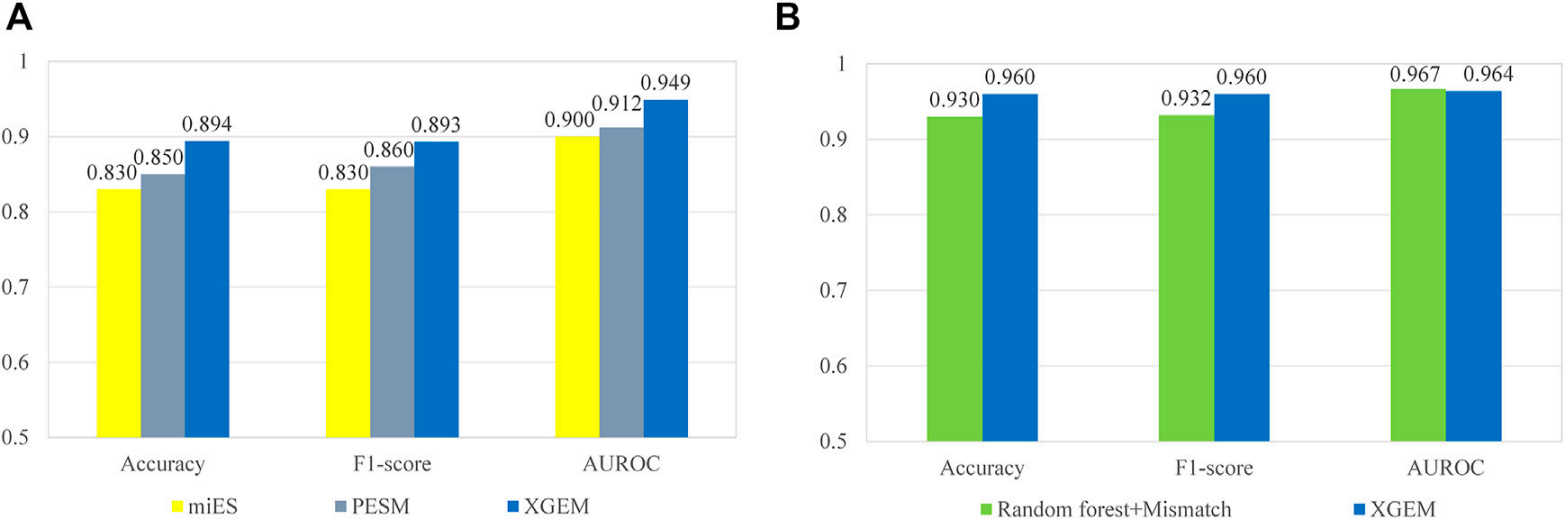
Comparison of different methods on mouse pre-miRNA datasets. The accuracy, F1-score, and AUROC are compared. **(A)** A comparison between the XGEM, miES, and PESM method on the miES-PESM dataset; **(B)** A comparison between XGEM and Ru’s work on Ru’s dataset.

The comparisons with miES and PESM were performed on the miES-PESM dataset. A 50-time repetition of 5-fold cross-validation was performed by all three methods on the same dataset. The repetition was used to eliminate inevitable randomness in the process of 5-fold cross-validation. The average performance values of the 50-time repetition were compared. The comparison with Ru’s work was performed on Ru’s dataset. Leave-one-out cross-validation was performed by both methods on the same dataset. The comparison details are depicted in Figure 6. XGEM performed the best in both comparisons. Although the benefits of XGEM is not large enough for us to claim that XGEM is definitely a better choice in predicting essential miRNAs, it is enough to state that XGEM is a better or at least comparable method to all state-of-the-art methods.

## Conclusion

Determining essentiality of non-coding genes is an important and fruitful research area, particularly for computational biology. In this article, we developed XGEM, which is a computational tool for predicting essential miRNAs. We evaluated the performance of XGEM in the mouse genome, with comparison to other state-of-the-art methods. The results indicated that XGEM has a potential to identify essential miRNAs. This is useful in understanding the biological functions of miRNA genes. We plan to establish a web server for hosting the implementation of XGEM. Due to the availability of limited resources currently, we will do this as a future work. In addition, the technology for developing XGEM can be extended to identify other types of essential non-coding genes, particularly those non-coding small RNA genes.

## Data Availability

Publicly available datasets were analyzed in this study. These data can be found here: https://github.com/minhui803/XGEM.
